# Pilot study of optical coherence tomography angiography-derived microvascular metrics in hands and feet of healthy and diabetic people

**DOI:** 10.1038/s41598-022-26871-y

**Published:** 2023-01-20

**Authors:** Gavrielle R. Untracht, Nikolaos Dikaios, Abdullah K. Durrani, Mariam Bapir, Marinko V. Sarunic, David D. Sampson, Christian Heiss, Danuta M. Sampson

**Affiliations:** 1 Department of Electrical, Electronic and Computer Engineering, The University of Western Australia, Perth, 6009 Australia; 2 School of Biosciences and Medicine, The University of Surrey, Guildford, GU27XH UK; 3 Mathematics Research Centre, Academy of Athens, Athens, 10679 Greece; 4 School of Physics, Advanced Technology Institute, The University of Surrey, Guildford, GU27XH UK; 5 Institute of Ophthalmology, University College London, London, EC1V 2PD UK; 6 Department of Medical Physics and Biomedical Engineering, University College London, London, WC1E 6BT UK; 7 East Surrey Hospital, Surrey and Sussex Healthcare NHS Trust, Redhill, RH15RH UK

**Keywords:** Biomarkers, Imaging and sensing

## Abstract

Optical coherence tomography angiography (OCTA) is a non-invasive, high-resolution imaging modality with growing application in dermatology and microvascular assessment. Accepted reference values for OCTA-derived microvascular parameters in skin do not yet exist but need to be established to drive OCTA into the clinic. In this pilot study, we assess a range of OCTA microvascular metrics at rest and after post-occlusive reactive hyperaemia (PORH) in the hands and feet of 52 healthy people and 11 people with well-controlled type 2 diabetes mellitus (T2DM). We calculate each metric, measure test–retest repeatability, and evaluate correlation with demographic risk factors. Our study delivers extremity-specific, age-dependent reference values and coefficients of repeatability of nine microvascular metrics at baseline and at the maximum of PORH. Significant differences are not seen for age-dependent microvascular metrics in hand, but they are present for several metrics in the foot. Significant differences are observed between hand and foot, both at baseline and maximum PORH, for most of the microvascular metrics with generally higher values in the hand. Despite a large variability over a range of individuals, as is expected based on heterogeneous ageing phenotypes of the population, the test–retest repeatability is 3.5% to 18% of the mean value for all metrics, which highlights the opportunities for OCTA-based studies in larger cohorts, for longitudinal monitoring, and for assessing the efficacy of interventions. Additionally, branchpoint density in the hand and foot and changes in vessel diameter in response to PORH stood out as good discriminators between healthy and T2DM groups, which indicates their potential value as biomarkers. This study, building on our previous work, represents a further step towards standardised OCTA in clinical practice and research.

## Introduction

There is growing evidence that structural changes and dysfunction of the microvasculature (the smallest vessels in the human body) in the skin and other organs are important biomarkers of risk and contributors to the pathophysiology of age-related diseases including diabetes and cardiovascular disease, the leading causes of death worldwide^1^. Specifically for skin, various imaging techniques have been used to characterise cutaneous microvascular structure and function; they are often limited to very specific regions (e.g., nailfold), require exogenous contrast agents, or have poor spatial resolution^2^. One technique stands out: optical coherence tomography angiography (OCTA). OCTA is potentially attractive to clinicians as it can be applied quickly (with acquisitions of typically less than 1 min without requiring specialized facilities), is non-invasive—not requiring administration of dye—and, distinctively, can provide high-resolution images of the microvascular network from different depths^3^. Although, originally developed for retinal imaging, the application of OCTA in cutaneous microvasculature imaging is growing^4,5^. OCTA has been used in a wide range of applications in dermatology, including: to monitor and assess wound healing^6–9^, visualise and quantify the response of microvasculature to applied pressure^10,11^, assess the structure and function of foot microvasculature of subjects with diabetes^12^, characterise microvasculature in basal cell carcinoma^13^, measure vessel pulsatility^14^, and assess the impact of ageing and blood pressure^2,15^. Although published reports demonstrate the feasibility of OCTA to support detection of cutaneous microvascular structure, dysfunction, and its response to treatment, they have largely focused on single metrics for microvascular network characterisation (usually vessel area density and/or mean vessel diameter), infrequently report test–retest repeatability, and often do not report the impact of demographic risk factors on microvascular structure and function.

The microvascular network represents a complex architecture. This complexity suggests that a variety of metrics may be needed to capture information on different aspects of microvascular structure and function under various conditions. For example, distinctive vessel morphology has been observed for a variety of skin tumours^16^. Further, a reduction in vessel area density has been seen for scleroderma^17^, eczema^18^, and scars^6^ undergoing treatment and a reduction in vessel area density and continuity was reported for vasculitis^19^. Widening of capillaries and increased tortuosity has been observed in plaque psoriaris^20^ and chronic venous disease^21^. Impaired vasodilation has been demonstrated for type 2 diabetes mellitus (T2DM), Raynaud’s phenomenon, and erythromelalgia^22,23^. Comprehensive characterisation of the microvascular network through multiple metrics will provide the strongest prospects for identifying the best biomarkers for future early diagnosis and monitoring of diseases and treatments^24^. Test variability is also an important parameter to define as it can be used to set a threshold for true changes in the OCTA microvascular metrics in longitudinal studies. Furthermore, it is crucial to understand how demographic risk factors impact the metrics and identify which metrics are best candidates to become biomarkers for diagnosis, monitoring, and treatment guidance of disease^23,25^.

To support the development of OCTA for assessment of cutaneous microvasculature towards the investigation of new biomarkers and future widespread clinical adoption, we present pilot data giving age-dependent and extremity-specific (hand vs foot) reference values for nine microvascular metrics: vessel area density, vessel length density, mean and median vessel diameter, mean and median vessel length, branchpoint density, fractal dimension, and mean tortuosity. All data were analysed with the open-source OCTAVA software previously developed by our group^26^. Images were acquired at baseline (to study microvascular structure) and after external stimuli (to study microvascular function) and the change in all nine metrics was assessed. Baseline measurements were taken five times to establish test–retest repeatability. We explored the effect of age, sex, imaging site, blood pressure, height, weight, body mass index (BMI), and health status (healthy versus well-controlled T2DM) on all microvascular metrics. This study directly builds on our previous work and is one step further towards the implementation of standardised OCTA in clinical practice and research.

## Methods

### Study design

This study was undertaken in the Clinical Research Facility, School of Biosciences and Medicine, The University of Surrey. All participants were asked to fast for at least 4 h prior to their scheduled imaging session and were asked to abstain from flavanol-rich foods, such as nuts, chocolate, tea, coffee, fruit, and vegetables, for at least 12 h prior to imaging. Imaging sessions were scheduled throughout the day. Height and weight were self-reported by participants during a pre-screening interview. Smoking habits, medications, and other factors which might impact circulation were also recorded. Participants rested in a supine position with legs straight and extended for 10 min before any measurements were taken and remained in this position until all imaging was completed. After the rest period, brachial blood pressure was measured 3 times using an automatic blood pressure cuff. Ankle brachial pressure index (ABPI) was measured to exclude participants with peripheral vascular disease (ABPI < 0.9). OCTA images were acquired, first on the hand and then on the foot. The hand was placed so that the height was even with the body and the arm was straight and fully extended and the legs remained straight and extended through the duration of the measurement. This study was approved to be undertaken through the issue of a favourable ethical opinion by *the University of Surrey Ethics Committee* (FHMS 19–20 060). Written, informed consent was obtained from all participants in adherence to the Declaration of Helsinki. This study is registered on clinicaltrials.gov (NCT04897191).

### OCTA imaging

OCTA images were acquired using the multi-beam VivoSight Dx (*Michelson Diagnostics Ltd*, Maidstone, Kent, UK). This instrument enables imaging of cutaneous microvasculature with a 20 kHz line-scan rate and an imaging resolution of 5.5 μm and 7.5 μm in the axial and transverse directions, respectively. It uses four beams of light, each focusing on a different depth, to improve resolution and provide deeper penetration.  The swept-wavelength source employed has a central wavelength of 1305 nm and a bandwidth of 140 nm. The total power at the sample is in the range of 5–10 mW. For all participants, the skin on the dorsum of the right hand, between the thumb and forefinger, and on the dorsum of the right hallux (big toe) was imaged. The handheld OCT probe was positioned for imaging on the skin through a plastic cap to reduce motion artefacts and maintain a constant distance between the imaging probe and the skin. Volumetric images were acquired over a 5 by 5 mm square area with spatial sampling set automatically by the imaging system of 4.4 μm along the fast axis and 41 μm along the slow axis. This corresponds to 1174 A-scans per B-scan and 120 B-scans per volume. Two repeated B-scans per location with an interscan time of 3.5 ms were acquired to generate angiograms. A black spot was drawn with a permanent marker on the skin to ensure repeated imaging of the same location and a blood pressure cuff was placed on the lower arm or calf of the participant for the hand and foot imaging, respectively, before commencing imaging. Five images were acquired sequentially at baseline conditions at the same location without adjusting the placement of the handheld probe (misalignment was observed between scans to be generally less than 5% (0.25 mm)). The cuff was inflated to 200 mmHg for 5 min to induce ischaemia. After 5 min, the pressure was released, and six sequential OCTA scans were acquired over a period of approximately 5 min to observe the post-occlusive reactive hyperaemia (PORH) response. 5 min after the onset of PORH is expected to be sufficient for the microvascular metrics to return to baseline values. Each OCTA scan required a duration of approximately 30 s, and the total measurement time per imaging site was approximately 15 min. We found this acquisition time was tolerated by the participants and resulted in minimal motion artefacts.

**Figure 1:**
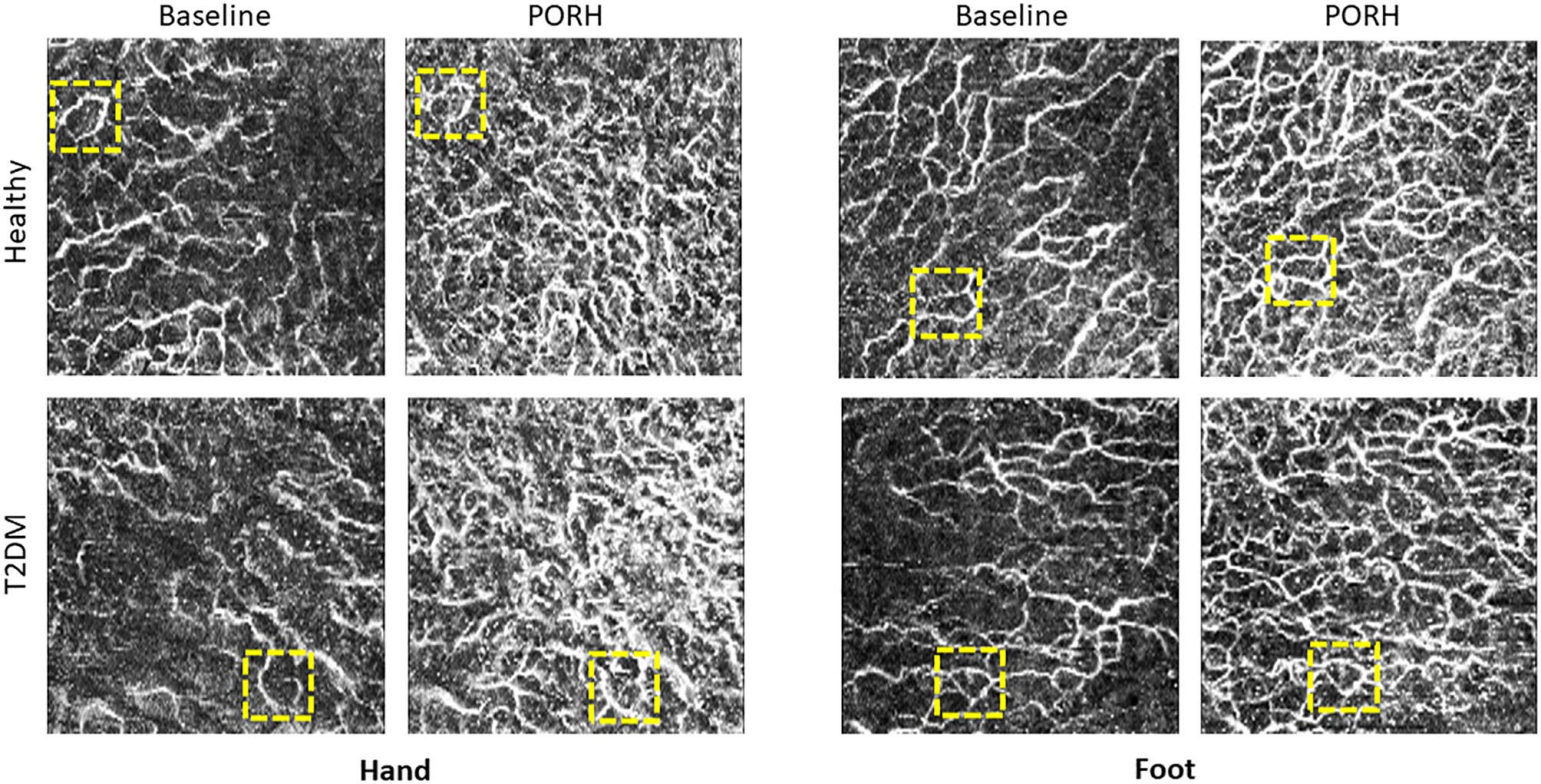
Representative OCTA MIP images from the hand (left) and the foot (right) representing the healthy group (top) and the diabetic group (bottom). All images are from participants in the 50–60 years old age group. All images are 5 × 5 mm. Dotted yellow boxes indicate 1 × 1 mm identical regions in the baseline and PORH datasets and demonstrate the amount of misalignment between scans (< 5% linear offset in x, y dimensions on average for all images).

Two-dimensional (2D) *en face* OCTA angiograms of the superficial plexus were generated in MATLAB 2020a (*The MathWorks, Inc.,* Natick, Massachusetts, USA*)* based on maximum intensity projection (MIP) of the volumetric angiogram over a physical thickness of 500 µm indepth with starting point 175 µm below the skin surface, assuming an average group refractive index of 1.4. The full procedure is explained in Ref.^26^. The same procedure was used for the hand and foot. Each final *en face* OCTA MIP image was inspected before undertaking quantitative analysis; images with significant artefacts or of poor quality were excluded. Examples of representative MIP images for a healthy and diabetic participant within the same age group are shown in Fig. 1. Quantitative metrics were extracted from the images using the OCTAVA software^26^. A description of the metrics evaluated in this study is presented in Table 1.

**Table 1:** A summary of the metrics evaluated in this study.

Metric	Unit	Description
Vessel area density (VAD)	%	Perfused blood vessel area in binarized OCTA MIP image divided by total image area
Vessel length density (VLD)	%	Total length of all observable vessels measured along the vessel centreline divided by the total image area. Length measurements are calculated from the skeletonized OCTA MIP image
Branchpoint density (BD)	Nodes/mm	Number of identified nodes divided by total vessel length
Mean diameter (MeanD)	μm	Mean diameter derived from a local thickness algorithm applied to single vessels in the binarized OCTA MIP. Mean diameter is the mean for a single vessel including one value for each identified segment
Median diameter (MedD)	μm	Median of single-vessel diameter measurements including one value for each identified segment
Mean tortuosity (MeanT)	–	Tortuosity evaluated from the skeletonized image using the arc length-over-chord ratio
Mean length (MeanL)	μm	Mean of the single-vessel centreline lengths including one value for each identified segment measured from the skeletonized image
Median length (MedL)	μm	Median of the single-vessel centreline lengths including one value for each identified segment measured from the skeletonized image
Fractal dimension (FD)	–	Indication of how the network fills space on variable length scales, calculated using the box counting method. FD is reported as FD ± FD_error_^27^

All metrics were calculated using the open-source software OCTAVA^26^.

### Sample size calculation

Power calculations were used to minimise the number of study participants required to demonstrate a statistical difference in quantitative measures. There is no published literature on a similar demographics study for direct comparison. However, previous OCTA literature has looked at detecting the differences in the microvascular vessel area density and mean diameter, at baseline and after external stimulus (local heating), between diabetic people with foot ulcer (diabetics with foot ulcer (DFU), HbA1c: 63 ± 14 mmol/mol), without foot ulcer (diabetics without foot ulcer (DNU), HbA1c: 68 ± 23 mmol/mol), and healthy controls^12^. Argarini et al. showed that, at baseline, vessel area density was statistically higher in DFU compared with a control group (21.9% vs 14.3%, p = 0.048). Furthermore, the local heating induced a significant increase in vessel area density in all groups (all p < 0.001), with smaller changes in vessel area density for the DFU group than for the DNU and control groups, respectively (44.7% vs 53.5% vs 56.5%). Based on these results, we sought to detect at least a 15% change in vessel area density; thus, a sample size of 10 was expected to provide a statistical power of 0.8 at alpha = 0.05 (G*Power V.3.1., University of Dusseldorf^28^). Accordingly, the 52 healthy study participants were divided into four age categories with n ≥ 10.

### Statistical analysis

Statistical analysis was undertaken in IBM SPSS statistics v27 (*IBM Corporation*, Chicago, IL, USA) with the significance level set at 0.05. For each metric calculated by OCTAVA, the mean of the five baseline measurements and the maximum of the six PORH measurements were used for further analysis. Data are reported as mean, standard deviation (SD) and range. Normality of data was assessed using a Shapiro–Wilk test. Metrics between groups were compared using one-way analysis of variance (ANOVA). The relative change between the two measurements was calculated as a percentage using the formula $$\Delta Metric= \frac{PORH-Baseline}{Baseline}\times 100\%$$. The repeatability of each metric was calculated based on the within-subject standard deviation ($${S}_{w})$$ method introduced by Bland and Altman^29^. For each study participant, the standard deviation of repeated measurements (baseline scans) was calculated and squared to obtain the variance. The square root of the average variance for all participants gives the measurement error, $${S}_{w}.$$ The coefficient of repeatability (CR) is defined as 2.77 $${S}_{w}$$ and 95% confidence intervals (CI) as: CR $$\pm 1.96\cdot ({S}_{w}/\sqrt{2n(m-1)}$$), where *n* is the number of participants and *m* the number of measurements for each participant^30^. Kendall’s Tau correlation analysis was used to study the correlation between the OCTA metrics and factors including age, sex, height, weight, BMI, and blood pressure. Kendal’s Tau analysis was used since, as a nonparametric analysis, it does not assume a linear relationship between the metrics and demographic data or normally distributed data. To check the ability to distinguish T2DM individuals from healthy, we first applied an unpaired t-test to T2DM and an age-matching healthy sub-group. An unpaired t-test was also used to assess differences in microvasculature between the hand and foot. A logistic regression model was used to analyse how well the OCTA metrics can differentiate T2DM from a group of age-matched healthy participants. The receiver operator characteristic area under the curve (ROC AUC) was calculated for each metric based on a leave-one-out analysis. For leave-one-out analysis, one participant’s data was excluded, and a model was generated from the remaining participants. The model was then tested on the excluded data to calculate a predictive probability. The process was repeated for all participants to generate a ROC for each metric. The ROC AUC was used to rank the metrics based on how well they distinguish images of T2DM and healthy people; we consider metrics with ROC AUC greater than 0.7 as acceptable discriminators. This value is generally accepted as a threshold for acceptable discrimination^31^.

## Results

### Study participants

52 healthy individuals and 11 individuals with well-controlled T2DM were enrolled in this study. Participants were pre-screened, and candidates who had been diagnosed with peripheral vascular disease or heart disease were excluded. All participants with T2DM had a glycated haemoglobin (HbA1c) level below 50 mmol/mol indicating well-controlled disease and had no foot ulcers or other wounds on their lower limbs. A description of the study participants is presented in Table 2.

**Table 2:** Clinical characteristics of study participants per health status and age group.

Study participants’ health status and healthy age group (years)	Healthy < 35	Healthy 35–49	Healthy 50–60	Healthy 61–76	T2DM
Number	14	14	11	13	11
Sex [F:M]	8:6	10:4	7:4	3:11	3:8
Age [years]	27 (4)	43 (4)	56 (2)	67 (6)	59 (10)
BMI [kg/m^2^]	25 (4)	22 (3)	25 (4)	25 (4)	30 (5)
Systolic blood pressure [mmHg]	118 (8)	113 (11)	121 (16)	125 (39)	132 (19)
Diastolic blood pressure [mmHg]	67 (11)	69 (8)	74 (11)	74 (12)	78 (11)
HbA1c [mmol/mol]	NA	NA	NA	NA	47 (4)

Values are expressed in mean (standard deviation).

*T2DM* type 2 diabetes mellitus, *F* female, *M* male, *BMI* body mass index, *HbA1c* glycated haemoglobin, *NA* not applicable.

### Age- and extremity-dependent reference values and coefficients of repeatability for OCTA microvascular metrics in healthy participants

**Figure 2:**
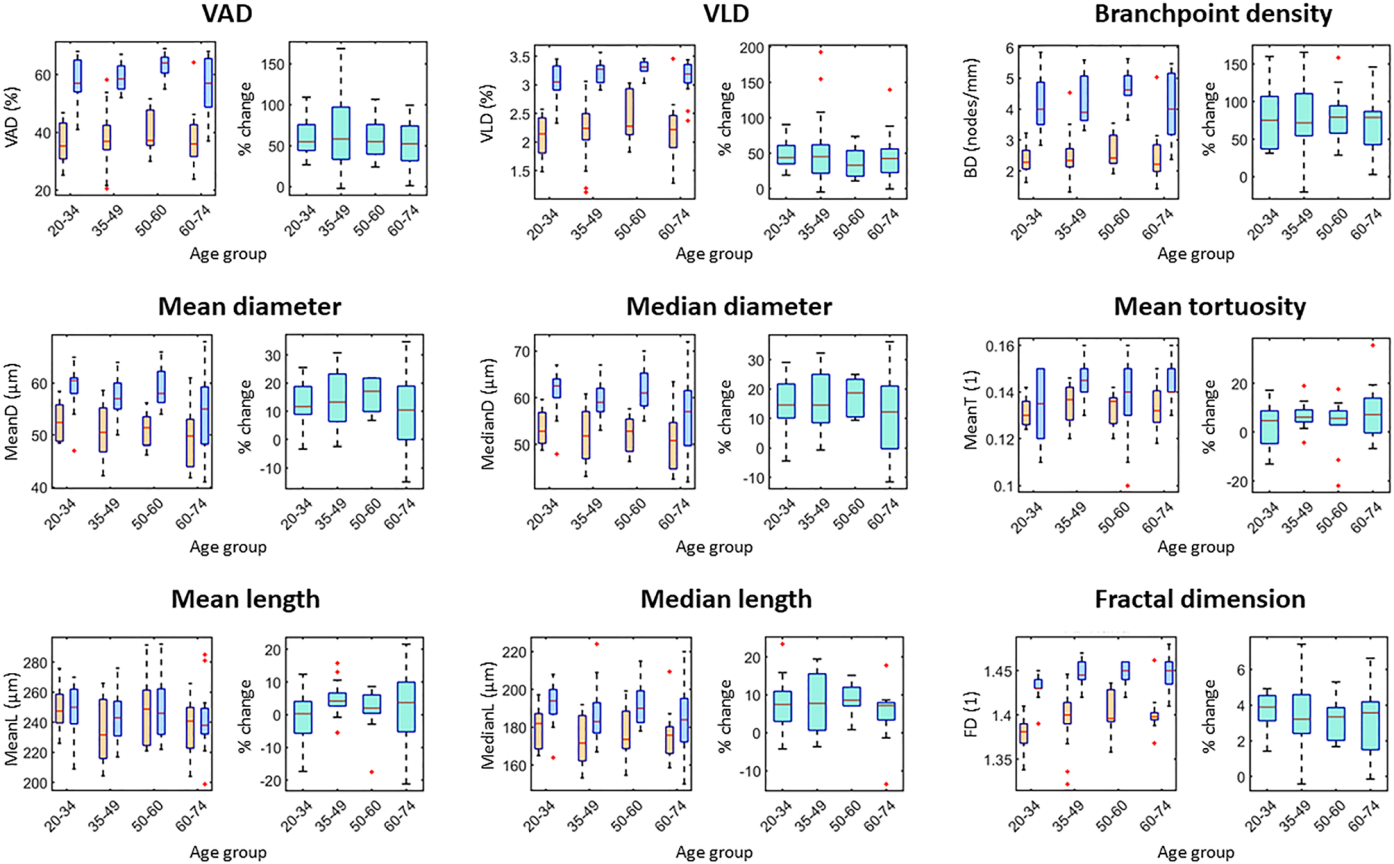
Box plots of age-dependent healthy reference values for selected metrics in the hand. For each metric, the left plot shows the values at baseline (orange) and PORH (blue) and the right plot shows the % change (turquoise). Red lines indicate the median value, boxes indicate the 25th and 75th percentiles, and the black dotted lines indicate the range. Outliers are indicated by red crosses. A unit of 1 indicates a dimensionless metric. *VAD* vessel area density, *VLD* vessel length density.

**Figure 3:**
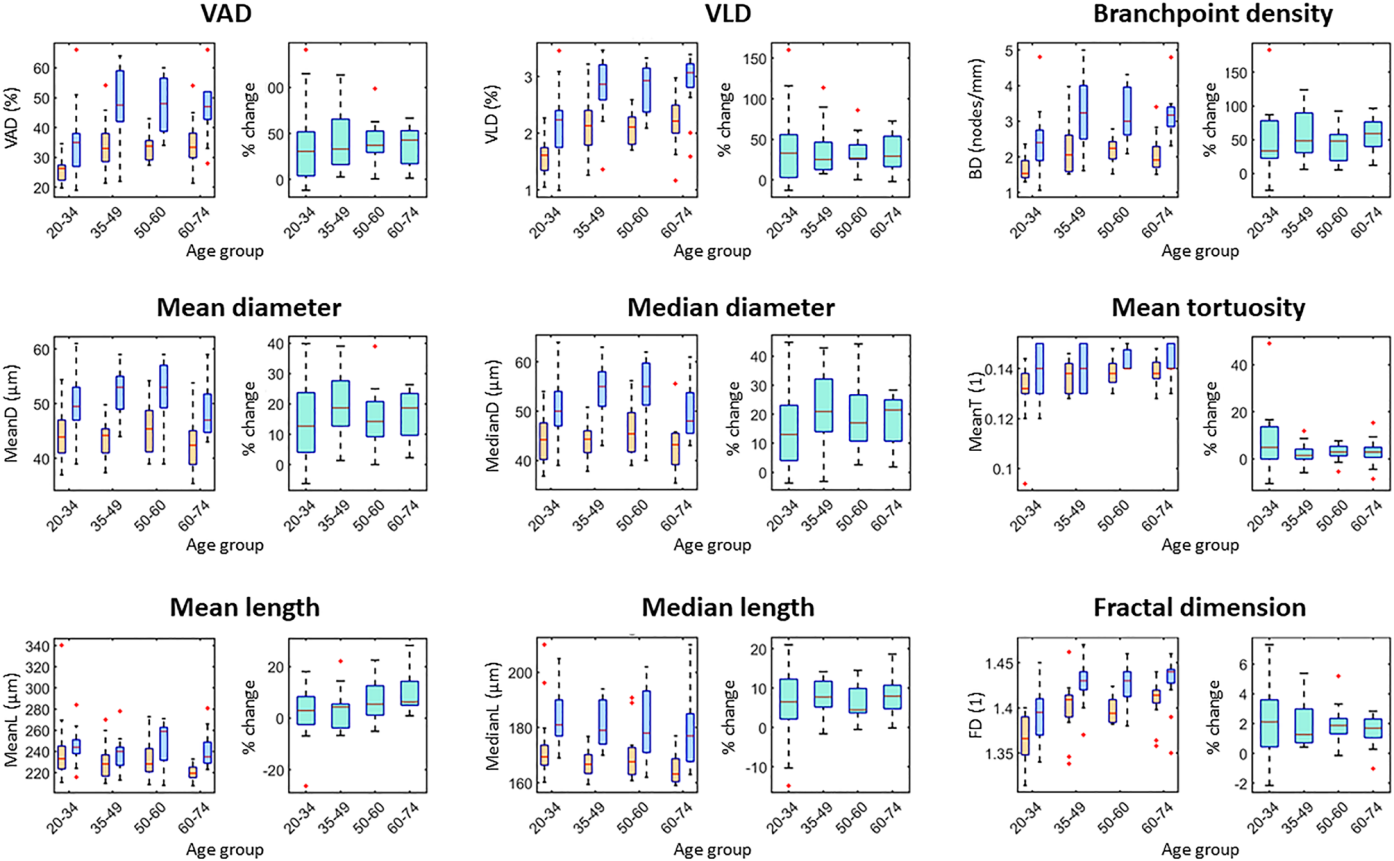
Box plots of age-dependent healthy reference values for selected metrics in the foot. For each metric, the left plot shows the values at baseline (orange) and PORH (blue) and the right plot shows the % change (turquoise). Red lines indicate the median value, boxes indicate the 25th and 75th percentiles, and the black dotted lines indicate the range. Outliers are indicated by red crosses. A unit of 1 indicates a dimensionless metric. *VAD* vessel area density, *VLD* vessel length density.

The healthy participants were divided into four age categories and OCTA microvascular metrics were generated at baseline and at the maximum of PORH (max PORH) thereby representing 18 metrics in total for each of hand and foot: 9 for baseline and 9 for max PORH. The results are summarized in Figs. 2 and 3 and a full list of results is available in Tables S1–S4. One-way ANOVA analysis showed no significant differences for age-dependent OCTA microvascular metrics in the hand, but significant differences for 9 out of 18 metrics in the foot (Tables S1, S2). Significant differences were observed between hand and foot for several metrics; in particular, for < 35-years-old and 50–60-years-old groups with generally higher values in hand (Table S3). There was notably a large variation within the groups for both extremities for most metrics. At the maximum of PORH, more vessels were visible, but the trends in each metric follow the same patterns as the baseline values. Overall, PORH significantly increased most of the OCTA microvascular metrics for hand and foot and for all age-groups. The test–retest variability of all the metrics is presented in Table S4. The coefficient of repeatability value ranges from 3.5 to 18% of the mean value for each metric, and coefficients of repeatability are generally lower than inter-individual variability for each metric. The coefficients of repeatability are better for foot than hand for all age-groups apart from the 61 to 76-years-old group.

### Correlation between microvascular metrics and demographic risk factors in healthy participants

To investigate the impact on OCTA metrics of factors such as age, sex, height, weight, body mass index (BMI), blood pressure, and imaging site, we performed a Kendall’s Tau correlation analysis. The results are summarized in Tables S5 and S6 (27 metrics in total for each of hand and foot: 9 for baseline, 9 for max PORH, and 9 for relative change between baseline and PORH). Considered alone, most of the factors that we evaluated were not significantly correlated with the OCTA-derived microvascular metrics. More of the metrics in the foot (10) were significantly correlated with age than metrics in the hand (6). Several metrics were more correlated with weight (6 in the hand and 7 in the foot)) or BMI (7 in the hand and 4 in the foot) than with age. 7 metrics in the hand and 2 in the foot were correlated with sex. Blood pressure was correlated with one microvascular metric in the hand, and with 4 metrics in the foot.

### OCTA microvascular metrics in well-controlled T2DM

To investigate how OCTA microvascular metrics differ between people with well-controlled T2DM and healthy people and to minimize the impact of other demographic factors, we selected for comparison 11 age-matched controls from our study cohort (Table S7). Overall, a significant difference was observed between the T2DM and age-matched controls for 6 microvascular metrics in hand and 6 in foot (Table S8). In the hand, significantly lower values were observed for VAD and BD at baseline and max PORH, as well as mean and median diameter at baseline between T2DM and healthy participants. However, the relative change between max PORH and baseline for VAD was higher for T2DM individuals. For foot, significantly lower values were observed for mean and median diameter at max PORH, and significantly higher values were seen for mean tortuosity at max PORH and fractal dimension at baseline between T2DM and healthy participants. The relative change between max PORH and baseline for mean and median diameters was significantly lower for T2DM individuals.

**Figure 4:**
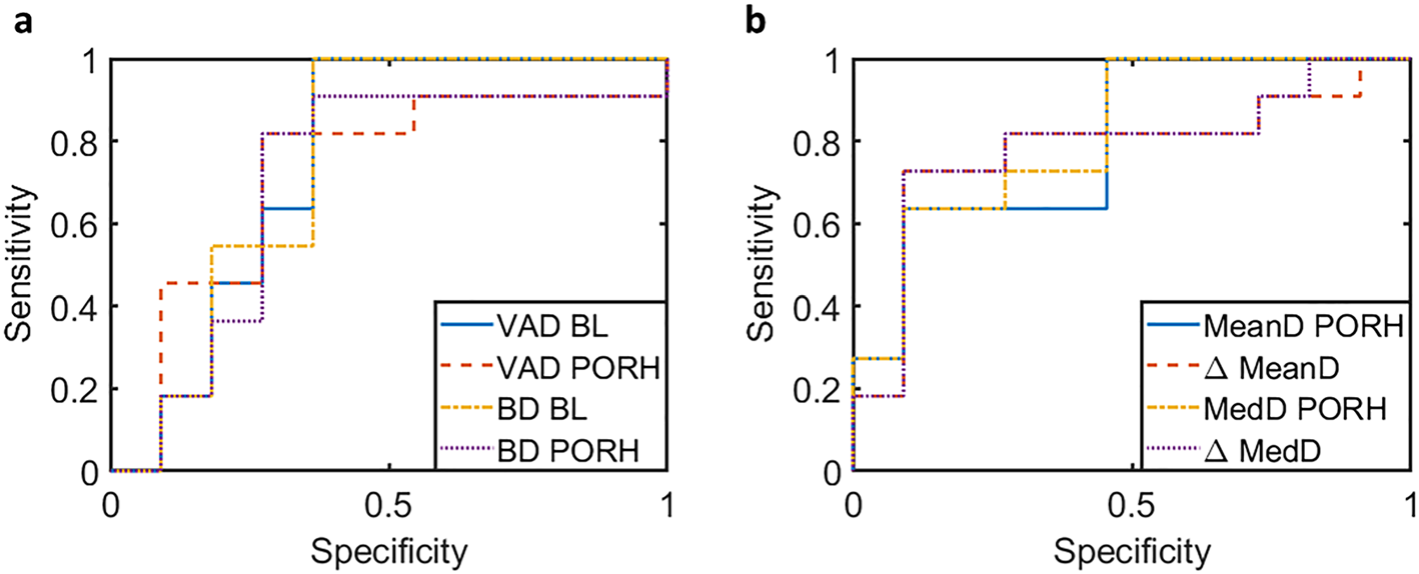
Representative ROC curves for selected metrics from the logistic regression analysis in the hand (**a**) and the foot (**b**). *VAD* vessel area density, *BD* branchpoint density, *MeanD* mean diameter, *MedD* median diameter, *PORH* post occlusive reactive hyperaemia, *BL* baseline. Area under the ROC curves for the metrics presented here are as follows (with 95% confidence interval): hand VAD BL—0.752 (0.528:0.977); hand VAD PORH—0.719 (0.487:0.951); hand BD BL—0.752 (0.528:0.977); hand BD PORH—0.702 (0.464:0.945); foot MeanD PORH—0.802 (0.613:0.990); foot ΔMeanD—0.785 (0.578:0.992); foot MedD PORH—0.818 (0.640:0.997); foot ΔMedD—0.777 (0.564:0.990).

To further investigate the differences in microvascular metrics between T2DM and healthy participants, we performed a logistic regression-based leave-one-out analysis using the same age-matched group of 11 T2DM and 11 healthy participants. This analysis evaluates which metrics change the most between healthy and T2DM individuals and, thus, which metrics can best distinguish between the two groups. The ROC AUC was calculated for each metric; a summary of the results is presented in Table S9. Representative ROC curves are shown for the hand (Fig. 4a) and foot (Fig. 4b) for selected metrics. In the hand, the baseline measurements generally tended to be better discriminators between the T2DM and healthy groups than the PORH or relative change. In the foot, the PORH and relative change of the mean diameter and median diameter were the most significant discriminators between the two groups. This observation suggests that change in vessel diameter in the foot in response to an external stimulus could be a promising biomarker that warrants further investigation.

## Discussion

Changes in cutaneous microvasculature have the potential to be a useful diagnostic tool in a range of diseases including T2DM, cardiovascular disease, hypertension, and Alzheimer’s disease^32,33^. Reference values, test–retest variability, and understanding of how various factors impact on OCTA-derived metrics are important. In this section, we discuss the clinical implications of our results and compare them with previously reported values. We comment on our study limitations and further investigations we believe are needed to support establishing OCTA as a clinical tool for cutaneous microvascular assessment.

### Metrics at baseline in healthy participants

Not all the metrics we have reported here have been previously investigated in skin but, for those metrics that have been previously reported, our values are readily comparable. The most reported metrics are VAD and mean diameter. Most reported values for VAD obtained using OCTA fall within the range 10–40% over a variety of skin sites^2,10,34–37^; the values measured during this study fall within this range (we measured an average VAD of 37% in the hand and 34% in the foot over all participants). Similarly, reported values for mean vessel diameter in the skin of the arm and leg lie in the range 31–70 µm. We measured a mean diameter of 50 µm in the hand and 40 µm in the foot. Both fractal dimension and tortuosity have been studied in the retina using OCTA, but only a few studies have reported values for fractal dimension in the skin, demonstrating wide variation^38,39^ and, to our knowledge, none for tortuosity.

The wide range of values for metrics reported in the literature is likely due to several factors. One consideration is the different skin sites chosen for imaging in the studies. The skin is a heterogeneous organ and the microvascular morphology varies between anatomical sites^40^. Second, OCTA instruments operate at different imaging speeds. This speed, along with scanning protocol chosen, determines the maximum flow velocity detectable by the OCTA system and, therefore, which vessels are visible in the OCTA image. Datasets with longer interscan times have better sensitivity to low flow velocities^25^. Longer interscan times can be achieved either by employing a slower scanning speed or by acquiring more scans at the same location to have access to data with varying interscan times. Finally, the image processing protocol, in particular, the segmentation algorithm and depth range for which the OCTA image is created, impacts on the generated metrics^41^. Thus, these factors make interpreting and comparing our results with others challenging.

It is important to mention that we observed a large variation within the age groups for both extremities for most metrics. Such variability is observed with other vascular metrics, such as flow-mediated dilation (FMD), is expected, and reflects the variability of healthy ageing phenotypes^42^. The threshold used in our sample size calculation was insufficient to account for the large demographic variability and heterogeneity in ageing phenotypes within the age groups. A larger study with less demographic variability within age groups could reduce the intra-group variability of OCTA metrics. Based on the average intra-group variability in this study over all the metrics, the group size should be scaled by a factor of 10 in future studies. This value was calculated using G*Power based on the mean and standard deviation of the microvascular metrics measured in this study. It is not surprising that quantifiable differences between healthy people of different ages are smaller than differences between healthy and well-controlled T2DM people since microvascular disease is a common complication of diabetes. As an additional benefit, a larger sample size would allow us to implement more sophisticated models such as a linear mixed model, dimensionality reduction, and machine learning-based models which require a large dataset to avoid overfitting. We also note that the selected imaging sites on the hand and the foot likely contribute to the large variability of metrics. When we originally designed the study, we chose to image the foot and the hand due to our interest in diabetic foot disease. However, as reported by Fuchs et al., the microcirculation in feet and toes (similarly to hands and fingers) fluctuates substantially within an individual subject due to the presence of arteriovenous anastomoses—which increase in prevalence closer to the extremities—and their response to external factors such as in thermoregulation^43^. This susceptibility to external factors suggests that the foot and the hand may not be the best skin sites to study ageing.

### Metrics after external stimulus in healthy participants

Several methods have been proposed for assessing cutaneous microvascular function including local^12^ or whole-limb heating^44^, application of local pressure^2,11^, and PORH through limb constriction^10^. Such measurements may be comparable to measurements of macrovascular endothelial function, which have proven to be an important biomarker of cardiovascular disease^45^. In all cases, the applied stimulus significantly increased OCTA microvascular diameter and VAD. Wang-Evers et al. have shown that local pressure stimulus significantly increased VAD (22.7% vs 30.7%—a 35% change) of the forearm in healthy individuals^2^. Argarini et al. have shown that local skin heating significantly increased OCTA microvascular diameter and VAD of the dorsal foot of healthy participants (53% change in diameter and 320% change in VAD)^12^. In another study, Argarini et al. have demonstrated that PORH significantly increased OCTA microvascular diameter (38 µm vs 45 µm—18% change) and vessel area density (5% vs 15–200% change) of the ventral forearm of healthy individuals^10^. In our study, we observe significant increase of OCTA microvascular diameter in the hand (51 µm vs 57 µm—11% change) and the foot (44 µm vs 51 µm—16% change), and VAD (hand: 38% vs 59%—55% change and foot: 32% vs 44%—38% change) in healthy participants. It is unclear whether the different external stimuli used by us and Argarini et al. measure the same functional response, or which would be most appropriately implemented in a clinical workflow considering a trade-off between clinical utility and complexity of the procedure. Some considerations to base a decision upon are measurement duration, repeatability, and patient tolerance. We found that only one participant was unable to tolerate the PORH measurement and the total measurement time was much less than reported for the heating-based measurement. More work is required to confirm the equivalence of these stimulation methods or otherwise—different stimuli and measurement protocols may probe different biological mechanisms that can be independently affected by different pathologies. However, the results of our study and others indicate that many more vessels are visible when a stimulus is applied; thus, such measurements after applied stimulus may be crucial for accurate measurement of microvascular structure to minimize uncertainty. This increase in vessel visibility can be due to a variety of factors: first, OCTA is only sensitive to perfused vessels within a specific range of flow speeds (as determined by the imaging speed and acquisition protocol). In baseline conditions, flow in capillary beds is inhomogeneous—but the flow rate and volume become more homogeneous in response to stimulus thereby providing the best opportunity to accurately map the full microvessel network^46^. In theory, this limitation could also be overcome by imaging at a much slower rate to observe the much slower perfusion rates in many vessels at rest, although this is not feasible with the system used in this study and increasing the scan time makes the measurement more susceptible to motion artefacts. Additionally, cutaneous microvasculature patency is highly dependent on environmental factors (such as room temperature), so response to an external stimulus can provide a more controlled protocol that can be applied in various settings without impacting the results.

### Test–retest repeatability

A high test–retest repeatability is an essential factor for distinguishing between normal variation of metrics and biomarkers of disease. Many studies have investigated test–retest variability of OCTA metrics in the retina, but only a few have assessed repeatability of imaging in skin^10,26,37^. Repeatability of OCTA imaging in the skin is impacted not only by day-to-day or environment-based variation in metrics, but also due to challenges of imaging the same location between imaging sessions or individual measurements. The comparability of metrics derived from images acquired at nearby (but not identical) locations on the skin has not been well studied. While imaging the same location on the skin, test–retest repeatability in each of these studies has shown that the intrasession variability is lower than the inter-individual variation in metrics—this implies that quantitative OCTA metrics can be used to detect physiological changes. Indeed, our results indicate that test–retest variability is similar among all age groups and less than the inter-individual variability; we measured a test–retest repeatability in the range 3.5–18% of the mean for all metrics. This has positive implications for the use of OCTA for longitudinal or follow-up studies of the same individual. In our study, we observed an offset of < 5% on average between sequential scans at the same location: the impact of this misalignment is quantified via the coefficients of repeatability that we report here. We did not control for variable pressure applied to the skin by the imaging probe in this study, although the operator used a notional protocol that ensured light, approximately constant pressure was applied; future measurements should include a pressure sensor between the imaging probe and the skin to ensure constant pressure is maintained between scans^47^.

While we did not measure intersession repeatability in this study, we note that longitudinal monitoring should be possible; Argarini et al. have measured an intersession coefficient of repeatability of 12% and 1.9%, for vascular area density and diameter, respectively^10^. Future studies assessing the impact of demographic factors should include several measurements of the same participants on different days to limit the impact of environmental factors on the results. Additionally, the variation between imaging at nearby skin sites should be quantified, as this could impact the coefficients of repeatability achieved when OCTA is implemented in a clinical setting. In general, coefficients of repeatability in the skin have been found to be worse than those for OCTA images in the retina^25^. This is a topic which warrants further study.

### Differences in metrics between the hand and foot

Previous studies have reported differences in the upper and lower limb vasculature in healthy people^48–50^ and with cardiovascular disease^51^. Donato et al. showed that during exercise, blood flow and conductance were attenuated in the legs of older healthy participants but not in the forearms. Beckman et al. reported attenuated vascular response in the lower limb of individuals with cardiovascular disease^51^. Imadojemu et al. have applied a tilt protocol (changing the body posture) and measured change in the brachial and femoral artery velocity before and after^52^. Their results showed greater vascular resistance in the leg than in the arm. Pawelczyk and Levine have studied limb response to infused adrenergic agonists and demonstrated greater response to a stimulus in the leg^53^. Comparing arms and legs in healthy participants, Newcomer et al. have shown smaller relative increases in blood flow and vascular conductance in the leg in response to an endothelium-dependent vasodilator^54^. The results of our study, which, to our knowledge, is the first study to use OCTA to investigate and compare the structure and function of the microvascular network of both hands and feet in T2DM and healthy  people, broadly agree with previously published reports using other measurement techniques. We observe a smaller relative increase in VAD (42% vs 55%; p = 0.440) and VLD (41% vs 48%, p = 0.709) but higher in mean (19% vs 11% change, p = 0.063), and median (22% vs 12%, p = 0.048) diameter in the foot compared to the hand in healthy participants, although the differences are not significant based on our sample size. A smaller but significant relative increase for VAD, VLD, and median diameter between foot and hand was visible for T2DM people,VAD (27% vs 62%; p = 0.030), VLD (26% vs 62%; p = 0.033), and median diameter (5% vs 15%; p = 0.038). Further study is necessary to better understand the exact mechanism responsible for age- and disease-related changes in various limbs and how to incorporate this knowledge into clinical practice^55,56^.

### Demographic factors impacting metrics

Some changes in microvasculature have been observed previously in response to ageing and other factors. It was previously reported that, in people over age 65, there is an increase in blood pressure and endothelial dysfunction that impacts on vessel stiffening and branching patterns^57^. Using video capillaroscopy, a decrease in capillary loop density by 40–70% and an increase in vascular length by 35–156% were observed comparing women in their 20’s to women in their 70’s^40^. Heiss et al. recently reported age-dependent reference values for FMD in the arm, demonstrating decreasing endothelial function with age^42^. Nishiyama et al. studied limb-specific effects of macrovascular structure and function by using FMD and Doppler ultrasonography on groups of 12 young (26 ± 2 years old) and 12 old (72 ± 1 years old) subjects. The authors showed age-related attenuation in leg FMD after normalization for shear rate (old 41% smaller FMD compared with young)^50^.

Hara et al. have observed changes in OCTA metrics associated with age^15^. However, they focused on facial imaging, a body region which is most exposed to the sun. Exposure to ultraviolet light in sunlight has already been linked to changes in the appearance and function of skin microvasculature and could, thus, explain why a correlation with age was observed in this study but not in others^40,58^. Recently, Wang-Evers et al. used OCTA to study microvascular structure and function in young (18–30 years old) and old (above 65 years old) study groups^2^. The authors demonstrated that baseline images did not show significant differences in OCTA metrics between age groups. Upon reactive hyperaemia, both groups demonstrated a significant increase within each metric. Similarly to Ref.^2^, we did not observe significant differences in OCTA-derived microvascular metrics at baseline associated with age in hand, but we did in foot. We did not see any age-related changes in the PORH response in hand, but we did in foot. The observed variability within each age group in our study indicates that age alone may not be the best way to categorize reference values for microvascular metrics.

Several studies have investigated the relationship between blood pressure and OCTA-derived metrics. A number of studies of retinal microvasculature have shown significant differences in vessel density in people with hypertension^59^. Guo et al. have observed a reduction in vessel density on the back of the hand in patients with hypertension (including baseline and after cold and warm stimulation)^60^. Wang-Evers et al. found no significant differences between OCTA metrics at baseline for people with hypertension (systolic blood pressure > 130 mmHg) vs normal blood pressure, but did observe a difference at PORH^2^. We did not observe any significant trends associated with blood pressure; however, we note that participants with diagnosed hypertension were excluded from our study.

The link between obesity and microvascular dysfunction has been well established^61–63^. Thus, it is not surprising that, among all the demographic risk factors considered in this study, BMI and weight had the strongest correlation with OCTA-derived metrics of microvasculature. However, based on our logistic regression analysis, several microvascular metrics were better discriminators between the healthy and T2DM groups than weight (ROC AUC = 0.645) or BMI (ROC AUC = 0.628). This indicates the potential for OCTA metrics as a diagnostic biomarker of diabetes in conjunction with these demographic factors. The role of obesity in other diseases, including diabetes and hypertension, is a topic of wide interest. Yet, to our knowledge, no studies have used OCTA to investigate cutaneous microvascular changes associated with obesity. This area could be an important one in finding potential microvascular biomarkers of metabolic diseases.

### Metrics in T2DM

Reduced local heating response in the skin of diabetic patients compared to healthy controls has been reported using laser Doppler flowmetry^43^. Recently, Argarini et al. have applied OCTA and local heating to investigate how microvascular structure and function differ between controls (CON), diabetic individuals without foot ulcer (DNU), and with foot ulcer (DFU)^12^. They reported higher microvascular density at baseline for DFU compared with CON (21.9% vs 14.3%; p = 0.048) and significant increase in diameter and density in all groups after local heating with smaller changes for the DFU group compared to CON. The relative change in mean diameter was 53%, 35.8% and 18.8% for CON, DNU and DFU, respectively. We see similar trends in the foot but also report values for the hand that, to the best of our knowledge, have not been reported yet. Similarly, we show that VAD in the foot is higher at baseline for T2DM compared to healthy participants (38% vs 32%; p = 0.075) and the relative change between baseline and after stimulus (in our case, PORH) is smaller for T2DM individuals (mean diameter: 5% vs 19% change, p = 0.003; change in VAD was not significant: VAD: 27% vs 42% change, p = 0.311). In contrast, VAD in the hand at baseline is higher for healthy compared to T2DM participants (VAD: 41% vs 32%, p = 0.036). Furthermore, we also report how other metrics vary, including VLD, BD, MeanT and FD change in T2DM and list metrics (Table S8) that are additional candidates to distinguish microvascular changes due to T2DM. In particular, branchpoint density at baseline and PORH show significant differences between healthy and T2DM groups in both the hand (BL: p = 0.034, PORH: p = 0.012) and the foot (BL: p = 0.034, PORH: p = 0.001). We note that the group of T2DM participants in our study had significantly lower HbA1c levels than the DNU group in Argarini et al. (47 ± 4 vs 63 ± 14 mmol/mol)^12^. The ability to detect differences between the healthy and T2DM groups in our study demonstrates the sensitivity of OCTA to small changes in microvascular structure and function. Based on the intra-group variability measured in this study, the power calculations indicate that group sizes of 10–20 participants will be sufficient to detect significant differences between groups. Due to the limited number of participants in the present study, we could not apply a multivariate approach to develop a model that would demonstrate to what extent the combination of several metrics enhances sensitivity of discrimination between people with T2DM and healthy people. This should be considered in future.

## Conclusions

This study builds directly on our previous work towards standardised OCTA in research and clinical practice. We define a wide range of microvascular metrics in the hand and the foot at baseline and the maximum of PORH in healthy participants and participants with well-controlled T2DM. The coefficient of repeatability is reported for each metric and our results show that coefficients of repeatability are generally lower than inter-individual variability indicating the applicability of OCTA for studies in larger cohorts, for longitudinal monitoring, and for assessing the efficacy of interventions. We show how metrics correlate with demographic and systemic risk factors; in particular, noting a correlation between microvascular metrics and BMI but lack of correlation with age for most metrics in healthy participants. Based on our results, we recommend a tenfold larger study to confirm age-related trends in microvascular metrics. We observed that several microvascular metrics, particularly branchpoint density in the hand and the foot and change in vessel diameter in the foot in response to PORH, were better discriminators between T2DM and healthy groups than any of the demographic factors considered in this study. These metrics should be further investigated as potential biomarkers to study T2DM microvascular complications in the hand and the foot. We also propose studing the impact on OCTA metrics of segmentation algorithms and the depth range for which the OCTA MIP is created, and undertaking a large-cohort investigation of OCTA-derived microvascular metrics in healthy and diseased people. These investigations will support the development of OCTA for the assessment of cutaneous microvasculature towards future widespread clinical adoption and the further investigation of new biomarkers.

## Supplementary Information

Supplementary Tables.

## Data Availability

The datasets generated during and/or analysed during the current study are available from the corresponding author on reasonable request.
