# Supplementary material for: Pilot study of optical coherence tomography angiography-derived microvascular metrics in hands and feet of healthy and diabetic people
Source: Sci Rep. 2023 Jan 20;13:1122. doi: 10.1038/s41598-022-26871-y (PMC9853488; doi:10.1038/s41598-022-26871-y)
Supplement: Supplementary file 1 — Supplementary Tables. [file 41598_2022_26871_MOESM1_ESM.docx]

|  | **Metric** | **<35yo (n=14)** | | **35-49yo (n=14)** | | **50-60yo (n=11)** | | **61-76yo (n=13)** | | **p-value** | **All together** | |
| --- | --- | --- | --- | --- | --- | --- | --- | --- | --- | --- | --- | --- |
|  |  | **Mean (SD)** | **95% CI** | **Mean (SD)** | **95% CI** | **Mean (SD)** | **95% CI** | **Mean (SD)** | **95% CI** |  | **Mean (SD)** | **95% CI** |
| **Hand** | VAD [%] BL | 36 (7) | 32-40 | 38 (11) | 32-45 | 41 (7) | 35-46 | 38 (10) | 32-44 | 0.615 | 38 (9) | 35-41 |
|  | VAD [%] PORH | 57 (8) | 53-62 | 60 (5) | 57-62 | 63 (4) | 60-66 | 56 (11) | 50-63 | 0.150 | 59 (8) | 57-61 |
|  | Mean ΔVAD [%] | 63 |  | 66 |  | 58 |  | 52 |  |  | 55 |  |
|  |  | | | | | | | | | |  |  |
|  | VLD [%] BL | 2.1 (0.4) | 1.9-2.3 | 2.2 (0.6) | 1.8-2.5 | 2.5  (0.4) | 2.2-2.8 | 2.2 (0.5) | 1.9-2.5 | 0.329 | 2.2 (0.5) | 2.1-2.4 |
|  | VLD [%] PORH | 3.1 (0.3) | 2.9-3.2 | 3.2 (0.2) | 3.1-3.3 | 3.3 (0.1) | 3.2-3.4 | 3.1 (0.3) | 2.9-3.3 | 0.123 | 3.2 (0.3) | 3.1-3.2 |
|  | Mean ΔVLD [%] | 50 |  | 57 |  | 38 |  | 47 |  |  | 45 |  |
|  |  | | | | | | | | | | | |
|  | BD [nodes/mm] BL | 2.4 (0.5) | 2.1-2.6 | 2.5 (0.8) | 2.0-3.0 | 2.7 (0.5) | 2.3-3.0 | 2.5 (0.9) | 2.0-3.1 | 0.777 | 2.5 (0.7) | 2.3-2.7 |
|  | BD [nodes/mm] PORH | 4.1 (1) | 3.5-4.7 | 4.3 (0.8) | 3.8-4.7 | 4.7 (0.6) | 4.3-5.1 | 4.1 (1.1) | 3.4-4.8 | 0.357 | 4.3 (0.9) | 4.0-4.5 |
|  | ΔBD [%] | 80 |  | 80 |  | 82 |  | 70 |  |  | 72 |  |
|  |  | | | | | | | | | |  |  |
|  | MeanD [μm] BL | 53 (3) | 51-55 | 51 (6) | 48-54 | 51 (3) | 49-53 | 49 (6) | 46-53 | 0.386 | 51 (5) | 50-52 |
|  | MeanD [μm] PORH | 59 (4) | 57-62 | 57 (4) | 55-59 | 59 (4) | 57-62 | 54 (8) | 49-59 | 0.063 | 57 (6) | 56-59 |
|  | Mean ΔMeanD [%] | 13 |  | 18 |  | 16 |  | 9 |  |  | 11 |  |
|  |  | | | | | | | | | |  |  |
|  | MedD [μm] BL | 53 (4) | 51-56 | 52 (6) | 49-56 | 52 (4) | 50-55 | 51 (6) | 47-54 | 0.580 | 52 (5) | 51-54 |
|  | MedD [μm] PORH | 61 (4) | 59-64 | 59 (4) | 57-62 | 61 (5) | 58-65 | 56 (9) | 51-62 | 0.111 | 60 (6) | 58-61 |
|  | Mean ΔMedD [%] | 15 |  | 19 |  | 18 |  | 11 |  |  | 15 |  |
|  |  | | | | | | | | | |  |  |
|  | MeanT [1] BL | 0.13 (0.01) | 0.13-0.14 | 0.13 (0.01) | 0.13-0.14 | 0.13 (0.01) | 0.13-0.14 | 0.13 (0.01) | 0.13-0.14 | 0.605 | 0.13 (0.01) | 0.13-0.14 |
|  | MeanT [1] PORH | 0.13 (0.01) | 0.13-0.14 | 0.14 (0.01) | 0.14-0.15 | 0.14 (0.02) | 0.12-0.15 | 0.14 (0.01) | 0.14-0.15 | 0.185 | 0.14 (0.01) | 0.14-0.14 |
|  | Mean ΔMeanT [%] | 2 |  | 11 |  | 3 |  | 8 |  |  | 8 |  |
|  |  | | | | | | | | | | | |
|  | MeanL [μm] BL | 250 (17) | 242-258 | 234 (25) | 222-246 | 247 (26) | 231-262 | 236 (23) | 224-248 | 0.107 | 241 (20) | 236-247 |
|  | MeanL [μm] PORH | 248 (17) | 238-258 | 244 (16) | 235-253 | 249 (21) | 235-263 | 241 (23) | 228-255 | 0.710 | 245 (19) | 240-251 |
|  | Mean ΔMeanL [%] | -1 |  | 5 |  | 1 |  | 3 |  |  | 2 |  |
|  |  | | | | | | | | | |  |  |
|  | MedL [μm] BL | 180 (14) | 173-186 | 173 (16) | 165-180 | 177 (17) | 168-186 | 176 (17) | 168-184 | 0.564 | 176 (13) | 173-180 |
|  | MedL [μm] PORH | 193 (12) | 186-199 | 186 (17) | 178-196 | 192 (13) | 184-201 | 185 (18) | 174-196 | 0.423 | 189 (15) | 185-193 |
|  | Mean ΔMedL [%] | 8 |  | 8 |  | 9 |  | 5 |  |  | 7.38 |  |
|  |  | | | | | | | | | |  |  |
|  | FD [1] BL | 1.38 (0.02) | 1.37-1.39 | 1.40 (0.03) | 1.38-1.42 | 1.40 (0.03) | 1.39-1.42 | 1.40 (0.02) | 1.39-1.41 | 0.153 | 1.40 (0.03) | 1.39-1.40 |
|  | FD [1] PORH | 1.43 (0.02) | 1.42-1.44 | 1.45 (0.02) | 1.44-1.45 | 1.45 (0.01) | 1.44-1.46 | 1.45 (0.02) | 1.43-1.46 | 0.064 | 1.44 (0.02) | 1.44-1.45 |
|  | Mean ΔFD [%] | 4 |  | 8 |  | 3 |  | 3 |  |  | 3 |  |

**Pilot study of optical coherence tomography angiography‐derived microvascular metrics in hands and feet of healthy and diabetic people**

**Supplemental tables**

**Table S1.** **Age-dependent reference values in hand for OCTA-derived metrics of healthy people at baseline (BL) and the maximum of post-occlusive reactive hyperaemia (PORH).** SD – standard deviation; 95% CI – 95% confidence intervals for the mean; p-value: Kruskal-Wallis test one-way analysis of variance; all p values > 0.05, so no significant differences between age categories were found; VAD – vessel area density; VLD – vessel length density; BD – branchpoint density; MeanD – mean diameter; MedD – median diameter; MeanT – mean tortuosity; MeanL – mean length; MedL – median length; FD – fractal dimension; [1] – dimensionless metric; fields highlighted in green show p<0.05 for paired t-test: baseline vs maximum PORH.

|  | **Metric** | **<35yo (n=14)** | | **35-49yo (n=14)** | | **50-60yo (n=11)** | | **61-76yo (n=13)** | | **p-value** | **All together** | |
| --- | --- | --- | --- | --- | --- | --- | --- | --- | --- | --- | --- | --- |
|  |  | **Mean (SD)** | **95% CI** | **Mean (SD)** | **95% CI** | **Mean (SD)** | **95% CI** | **Mean (SD)** | **95% CI** |  | **Mean (SD)** | **95% CI** |
| **Foot** | VAD [%] BL | 26 (4) | 23-29 | 34 (9) | 29-39 | 34 (5) | 30-37 | 35 (8) | 30-40 | **0.005** | 32 (8) | 30-34 |
|  | VAD [%] PORH | 36 (12) | 28-43 | 48 (12) | 41-55 | 48 (9) | 41-54 | 47 (9) | 41-52 | **0.011** | 44 (12) | 41-47 |
|  | Mean ΔVAD [%] | 37 |  | 43 |  | 42 |  | 36 |  |  | 38 |  |
|  |  | | | | | | | | | |  |  |
|  | VLD [%] BL | 1.6 (0.4) | 1.4-1.8 | 2.1 (0.6) | 1.8-2.4 | 2.1 (0.3) | 1.9-2.3 | 2.2 (0.5) | 1.9-2.5 | **0.002** | 2.0 (0.5) | 1.9-2.1 |
|  | VLD [%] PORH | 2.2 (0.7) | 1.8-2.6 | 2.8 (0.5) | 2.5-3.1 | 2.8 (0.5) | 2.5-3.1 | 2.9 (0.5) | 2.6-3.2 | **0.005** | 2.7 (0.6) | 2.5-2.8 |
|  | Mean ΔVLD [%] | 41 |  | 37 |  | 36 |  | 34 |  |  | 35 |  |
|  |  | | | | | | | | | | | |
|  | BD [nodes/mm] BL | 1.7 (9.3) | 1.5-1.8 | 2.2 (0.7) | 1.8-2.6 | 2.2 (0.4) | 1.9-2.4 | 2.1 (0.5) | 1.8-2.4 | **0.025** | 2.0 (0.6) | 1.9-2.2 |
|  | BD [nodes/mm] PORH | 2.5 (0.9) | 1.9-3.0 | 3.4 (1.0) | 2.8-4.0 | 3.2 (0.8) | 2.6-3.7 | 3.2 (0.6) | 2.8-3.6 | **0.037** | 3.0 (0.9) | 2.8-3.3 |
|  | Mean ΔBD [%] | 50 |  | 58 |  | 44 |  | 56 |  |  | 50 |  |
|  |  | | | | | | | | | |  |  |
|  | MeanD [μm] BL | 44 (5) | 41-47 | 44 (4) | 42-46 | 45 (5) | 42-49 | 42 (5) | 39-45 | 0.389 | 44 (5) | 43-45 |
|  | MeanD [μm] PORH | 50 (5) | 47-53 | 52 (5) | 50-55 | 52 (6) | 49-56 | 49 (5) | 46-52 | 0.238 | 51 (5) | 49-52 |
|  | Mean ΔMeanD [%] | 13 |  | 20 |  | 16 |  | 16 |  |  | 16 |  |
|  |  | | | | | | | | | |  |  |
|  | MedD [μm] BL | 45 (5) | 42-48 | 44 (4) | 42-47 | 46 (5) | 43-50 | 43 (5) | 40-46 | 0.410 | 44 (5) | 43-46 |
|  | MedD [μm] PORH | 51 (6) | 47-54 | 54 (6) | 51-58 | 55 (6) | 50-59 | 50 (6) | 47-53 | 0.409 | 52 (6) | 51-54 |
|  | Mean ΔMedD [%] | 14 |  | 22 |  | 19 |  | 18 |  |  | 18 |  |
|  |  | | | | | | | | | |  |  |
|  | MeanT [1] BL | 0.13 (0.01) | 0.12-0.14 | 0.14 (0.01) | 0.13-0.14 | 0.14 (0.01) | 0.13-0.14 | 0.14 (0.01) | 0.14-0.14 | **0.028** | 0.14 (0.01) | 0.13-0.14 |
|  | MeanT [1] PORH | 0.14 (0.01) | 0.13-0.14 | 0.14 (0.01) | 0.13-0.14 | 0.14 (0.01) | 0.14-0.15 | 0.14 (0.01) | 0.14-0.15 | 0.551 | 0.14 (0.01) | 0.14-0.14 |
|  | Mean ΔMeanT [%] | 8 |  | 2 |  | 3 |  | 3 |  |  | 3 |  |
|  |  | | | | | | | | | | | |
|  | MeanL [μm] BL | 242 (37) | 223-260 | 230 (21) | 220-240 | 234 (23) | 221-246 | 220 (13) | 216-225 | 0.095 | 231 (22) | 225-238 |
|  | MeanL [μm] PORH | 244 (17) | 235-254 | 238 (16) | 229-247 | 249 (20) | 235-263 | 241 (17) | 231-252 | 0.471 | 243 (17) | 238-248 |
|  | Mean ΔMeanL [μm] | 4 |  | 5 |  | 8 |  | 10 |  |  | 5 |  |
|  |  | | | | | | | | | |  |  |
|  | MedL [μm] BL | 174 (18) | 166-182 | 167 (10) | 164-170 | 170 (13) | 163-177 | 164 (9) | 161-168 | 0.090 | 169 (10) | 166-172 |
|  | MedL [μm] PORH | 183 (10) | 177-189 | 181 (8) | 176-185 | 181 (13) | 172-190 | 179 (12) | 171-186 | 0.767 | 181 (11) | 178-184 |
|  | Mean ΔMedL [%] | 7 |  | 9 |  | 6 |  | 9 |  |  | 7 |  |
|  |  | | | | | | | | | |  |  |
|  | FD [1] BL | 1.36 (0.03) | 1.35-1.38 | 1.40 (0.03) | 1.38-1.41 | 1.40 (0.01) | 1.41-1.44 | 1.41 (0.02) | 1.41-1.45 | **<0.001** | 1.39 (0.03) | 1.37-1.41 |
|  | FD [1] PORH | 1.39 (0.03) | 1.37-1.41 | 1.43 (0.02) | 1.41-1.44 | 1.43 (0.02) | 1.41-1.44 | 1.43 (0.03) | 1.41-1.45 | **0.002** | 1.42 (0.03) | 1.41-1.43 |
|  | Mean ΔFD [%] | 2 |  | 2 |  | 2 |  | 2 |  |  | 2 |  |

**Table S2.** **Age-dependent reference values in the foot for OCTA-derived metrics of healthy people at the baseline (BL) and the maximum of post-occlusive reactive hyperaemia (PORH).** SD – standard deviation; 95% CI – 95% confidence intervals for the mean; p-value: Kruskal-Wallis test one-way analysis of variance; **bolded** p<0.05; VAD – vessel area density; VLD – vessel length density; BD – branchpoint density; MeanD – mean diameter; MedD – median diameter; MeanT – mean tortuosity; MeanL – mean length; MedL – median length; FD – fractal dimension; [1] – dimensionless metric; fields highlighted in green show p<0.05 for paired t-test: baseline vs maximum PORH.

|  | **<35yo (n=14)** | **35-49yo (n=14)** | **50-60yo (n=11)** | **61-76yo (n=13)** | **All together** |
| --- | --- | --- | --- | --- | --- |
|  | **Mean Diff. (SD)** | **Mean Diff. (SD)** | **Mean Diff. (SD)** | **Mean Diff. (SD)** | **Mean Diff. (SD)** |
| VAD [%] BL | +10 (7) | +4 (11) | +7 (8) | +3 (14) | +6 (10) |
| VAD [%] PORH | +22 (17) | +12 (1) | +15 (9) | +10 (12) | +15 (13) |
| VLD [%] BL | +0.51 (0.38) | +0.07 (0.73) | +0.37 (0.54) | +0.02 (0.82) | +0.24 (0.65) |
| VLD [%] PORH | +0.90 (0.85) | +0.42 (0.54) | +0.50 (0.40) | +0.22 (0.63) | +0.52 (0.67) |
| BD [nodes/mm] BL | +0.70 (0.55) | +0.33 (0.79) | +0.46 (0.59) | +0.41 (1.06) | +0.48 (0.77) |
| BD [nodes/mm] PORH | +1.67 (1.39) | +0.91 (1.01) | +1.54 (0.82) | +0.91 (1.04) | +1.25 (1.13) |
| MeanD [μm] BL | +8 (5) | +7 (5) | +6 (6) | +7 (5) | +7 (5) |
| MeanD [μm] PORH | +10 (6) | +5 (6) | +7 (7) | +5 (5) | +7 (6) |
| MedD [μm] BL | +9 (5) | +8 (5) | +6 (7) | +8 (6) | +8 (6) |
| MedD [μm] PORH | +11 (6) | +5 (7) | +7 (8) | +6 (6) | +7 (7) |
| MeanT [1] BL | +0.001 (0.013) | -0.002 (0.009) | -0.005 (0.007) | -0.007 (0.009) | -0.003 (0.01) |
| MeanT [1] PORH | -0.005 (0.013) | +0.0042 (0.011) | -0.005 (0.016) | 0 (0.0138) | -0.001 (0.014) |
| MeanL [μm] BL | +8 (34) | +4 (21) | +13 (21) | +16 (21) | +10 (25) |
| MeanL [μm] PORH | +4 (21) | +6 (19) | 0 (29) | 0 (14) | +3 (21) |
| MedL [μm] BL | +6 (12) | +6 (12) | +7 (14) | +11 (12) | +7 (12) |
| MedL [μm] PORH | +10 (13) | +6 (18) | +11 (16) | +6 (12) | +8 (15) |
| FD [1] BL | +0.02 (0.03) | -0.01 (0.04) | +0.01 (0.03) | -0.01 (0.03) | 0 (0.03) |
| FD [1] PORH | + 0.04 (0.03) | +0.04 (0.04) | +0.02 (0.02) | +0.02 (0.03) | +0.03 (0.03) |

**Table S3.** **Results of paired sample t-test analysis to show age-dependent differences between hand and foot in healthy people**. Mean Diff – mean difference between hand and foot; SD – standard deviation; fields highlighted light green p<0.05, fields highlighted dark green p<0.001.

| **Imaging site** | **Metric** | **<35yo (n=14)** | | **35-49yo (n=14)** | | **50-60yo (n=11)** | | **61-76yo (n=13)** | | **All together** | |
| --- | --- | --- | --- | --- | --- | --- | --- | --- | --- | --- | --- |
|  |  | CR | CI | CR | CI | CR | CI | CR | CI | CR | CI |
| **Hand** | VAD [%] | 6.1 | 5.7-6.5 | 6.3 | 5.9-6.7 | 6.2 | 5.8-6.7 | 5.5 | 5.1-5.9 | 6.1 | 5.9-6.3 |
|  | VLD [%] | 1.6 | 1.5-1.7 | 1.6 | 1.5-1.7 | 1.5 | 1.4-1.6 | 1.4 | 1.3-1.5 | 1.5 | 1.5-1.6 |
|  | MeanD [μm] | 4.4 | 4.1-4.7 | 4.4 | 4.1-4.7 | 4.7 | 4.3-5.0 | 4.6 | 4.3-4.9 | 4.6 | 4.4-4.7 |
|  | MedD [μm] | 4.5 | 4.2-4.8 | 4.6 | 4.3-4.9 | 4.9 | 4.6-5.3 | 5.0 | 4.6-5.3 | 4.8 | 4.6-5.0 |
|  | MeanL [μm] | 9.3 | 8.6-9.9 | 9.9 | 9.2-10.5 | 10.0 | 9.2-10.8 | 9.7 | 9.1-10.4 | 9.7 | 9.4-10.0 |
|  | MedL [μm] | 8.5 | 7.9-9.0 | 8.3 | 7.8-8.9 | 8.9 | 8.2-9.5 | 9.2 | 8.6-9.9 | 8.7 | 8.4-9.0 |
|  | BD [nodes/mm] | 1.6 | 1.5-1.7 | 1.6 | 1.5-1.7 | 1.8 | 1.6-1.9 | 1.5 | 1.4-1.6 | 1.6 | 1.6-1.7 |
|  | MeanT [1] | 0.2 | 0.2-0.2 | 0.3 | 0.2-0.3 | 0.2 | 0.2-0.2 | 0.2 | 0.2-0.2 | 0.2 | 0.2-0.2 |
|  | FD [1] | 0.3 | 0.3-0.4 | 0.3 | 0.3-0.3 | 0.3 | 0.3-0.4 | 0.3 | 0.3-0.3 | 0.3 | 0.3-0.3 |
| **Foot** | VAD [%] | 5.5 | 5.1-5.9 | 5.4 | 5.1-5.8 | 5.7 | 5.3-6.2 | 5.8 | 5.4-6.2 | 5.6 | 5.4-5.8 |
|  | VLD [%] | 1.5 | 1.4-1.6 | 1.4 | 1.3-1.5 | 1.4 | 1.3-1.6 | 1.5 | 1.4-1.6 | 4.5 | 1.4-1.5 |
|  | MeanD [μm] | 4.8 | 4.4-5.1 | 4.1 | 3.9-4.4 | 4.3 | 4.0-4.6 | 4.5 | 4.2-4.9 | 4.4 | 4.3-4.6 |
|  | MedD [μm] | 5.1 | 4.7-5.4 | 4.4 | 4.1-4.7 | 4.7 | 4.3-5.0 | 4.7 | 4.3-5.0 | 4.7 | 4.5-4.9 |
|  | MeanL [μm] | 11.6 | 10.8-12.4 | 9.3 | 8.7-9.9 | 10.2 | 9.4-10.9 | 8.7 | 8.1-9.3 | 10.0 | 9.7-10.4 |
|  | MedL [μm] | 9.5 | 8.9-10.2 | 8.4 | 7.8-9.0 | 8.0 | 7.4-8.6 | 7.3 | 6.8-7.78 | 8.4 | 8.1-8.6 |
|  | BD [nodes/mm] | 1.6 | 1.5-1.7 | 1.5 | 1.4-1.6 | 1.6 | 1.5-1.7 | 1.6 | 1.5-1.7 | 1.6 | 1.5-1.6 |
|  | MeanT [1] | 0.3 | 0.3-0.3 | 0.3 | 0.2-0.3 | 0.3 | 0.2-0.3 | 0.3 | 0.3-0.3 | 0.3 | 0.3-0.3 |
|  | FD [1] | 0.4 | 0.3-0.4 | 0.3 | 0.3-0.3 | 0.3 | 0.3-0.3 | 0.3 | 0.3-0.3 | 0.3 | 0.3-0.3 |

**Table S4.** **Age-dependent test-retest repeatability of OCTA microvascular metrics for hand and foot in healthy people.** CR – coefficient of repeatability; CI – confidence interval; SD – standard deviation; VAD – vessel area density; VLD – vessel length density; MeanD – mean diameter; MedD – median diameter; MeanL – mean length; MedL – median length; BD – branchpoint density; MeanT – mean tortuosity; FD – fractal dimension; [1] – dimensionless metric.

|  | **Metric** | **Sex** | **Age** | **Height** | **Weight** | **BMI** | **BP Systolic** | **BP Diastolic** |
| --- | --- | --- | --- | --- | --- | --- | --- | --- |
| **Hand** | VAD BL | -0.094 | -0.052 | -0.026 | -0.159 | -0.190 | -0.037 | 0.004 |
|  | VAD PORH | -0.226 | -0.095 | -0.006 | -0.217 | -0.291 | -0.103 | -0.035 |
|  | ΔVAD | -0.031 | -0.033 | 0.062 | 0.035 | 0.013 | -0.082 | -0.066 |
|  |  | | | | | | | |
|  | VLD BL | -0.075 | -0.041 | -0.03 | -0.115 | -0.154 | -0.043 | 0.016 |
|  | VLD PORH | -0.059 | 0.001 | 0.081 | -0.082 | -0.194 | -0.027 | 0.032 |
|  | ΔVLD [%] | 0.086 | -0.024 | 0.097 | 0.126 | 0.111 | 0.004 | -0.032 |
|  |  | | | | | | | |
|  | MeanD BL | -0.194 | -0.210 | -0.106 | -0.166 | -0.147 | -0.068 | 0 |
|  | MeanD PORH | -0.257 | -0.276 | -0.146 | -0.224 | -0.168 | -0.109 | -0.032 |
|  | ΔMeanD | -0.114 | -0.033 | -0.065 | -0.07 | -0.024 | -0.003 | -0.009 |
|  |  | | | | | | | |
|  | MedD BL | -0.197 | -0.199 | -0.11 | -0.186 | -0.167 | -0.078 | 0.004 |
|  | MedD PORH | -0.257 | -0.266 | -0.135 | -0.223 | -0.171 | -0.094 | -0.02 |
|  | ΔMedD | -0.121 | -0.032 | -0.062 | -0.06 | -0.006 | 0.004 | 0.001 |
|  |  | | | | | | | |
|  | MeanL BL | -0.11 | -0.083 | -0.08 | -0.071 | -0.026 | -0.006 | 0.046 |
|  | MeanL PORH | -0.051 | -0.051 | 0.017 | -0.002 | 0.051 | 0.138 | 0.165 |
|  | ΔMeanL | 0.075 | 0.078 | 0.093 | 0.027 | -0.008 | 0.067 | 0.06 |
|  |  | | | | | | | |
|  | MedL BL | -0.217 | -0.081 | -0.156 | -0.148 | -0.092 | -0.066 | 0.03 |
|  | MedL PORH | -0.117 | -0.128 | 0.012 | -0.039 | -0.062 | 0 | 0.098 |
|  | ΔMedL | 0.049 | -0.047 | 0.133 | 0.071 | 0.017 | 0.1 | 0.11 |
|  |  | | | | | | | |
|  | BD BL | -0.083 | -0.07 | -0.031 | -0.143 | -0.170 | -0.046 | -0.003 |
|  | BD PORH | -0.227 | -0.07 | 0.009 | -0.199 | -0.272 | -0.073 | -0.03 |
|  | ΔBD | -0.132 | -0.05 | 0.069 | -0.027 | -0.063 | -0.039 | -0.007 |
|  |  | | | | | | | |
|  | MeanT BL | 0.093 | -0.003 | 0.056 | -0.005 | -0.044 | 0.017 | -0.02 |
|  | MeanT PORH | 0.058 | 0.115 | 0.015 | -0.05 | -0.076 | -0.117 | -0.158 |
|  | ΔMeanT | 0.009 | 0.141 | 0.002 | 0.03 | 0.034 | -0.092 | -0.107 |
|  |  | | | | | | | |
|  | FD BL | 0.03 | 0.096 | 0.034 | -0.075 | -0.122 | 0.009 | -0.002 |
|  | FD PORH | -0.023 | 0.133 | 0.114 | -0.104 | -0.231 | -0.059 | -0.034 |
|  | ΔFD | -0.048 | -0.068 | 0.037 | 0.037 | -0.005 | -0.078 | -0.06 |
|  |  | | | | | | | |
|  | FDerror BL | 0.253 | 0.251 | 0.172 | 0.234 | 0.213 | 0.129 | 0.085 |
|  | FDerror PORH | 0.274 | 0.334 | 0.099 | 0.155 | 0.155 | 0.243 | 0.164 |
|  | ΔFDerror | -0.004 | 0.1 | -0.118 | -0.108 | -0.089 | 0.094 | 0.044 |
|  |  | | | | | | | |
|  | Target | 0.202 | 0.280 | 0.076 | 0.320 | 0.359 | 0.221 | 0.2 |
|  |  | | | | | | | |

**Table S5.** **Kendall Tau correlation coefficients between demographic risk factors and microvascular metrics for hand in healthy people**. VAD – vessel area density; VLD – vessel length density; MeanD – mean diameter; MedD – median diameter; MeanL – mean length; MedL – median length; BP – branchpoint density; MeanT – mean tortuosity; FD – fractal dimension; BL – baseline; PORH – post-occlusive reactive hyperaemia; Light green shading – correlation is significant at the 0.05 level (2-tailed); Dark green shading – correlation is significant at the 0.01 level (2-tailed).

|  | **Metric** | **Sex** | **Age** | **Height** | **Weight** | **BMI** | **BP Systolic** | **BP Diastolic** |
| --- | --- | --- | --- | --- | --- | --- | --- | --- |
| **Foot** | VAD BL | 0.08 | 0.244 | -0.026 | -0.08 | -0.101 | 0.032 | 0.04 |
|  | VAD PORH | -0.008 | 0.138 | -0.094 | -0.135 | -0.133 | -0.07 | -0.062 |
|  | ΔVAD | -0.062 | 0.031 | -0.037 | -0.054 | -0.048 | -0.11 | -0.114 |
|  |  | | | | | | | |
|  | VLD BL | 0.092 | 0.258 | -0.053 | -0.074 | -0.077 | 0.029 | 0.015 |
|  | VLD PORH | 0.022 | 0.167 | -0.06 | -0.101 | -0.099 | -0.037 | -0.05 |
|  | ΔVLD | -0.037 | -0.005 | 0.012 | 0.017 | -0.002 | -0.05 | -0.09 |
|  |  | | | | | | | |
|  | MeanD BL | -0.036 | -0.145 | -0.022 | -0.031 | -0.01 | 0.056 | 0.077 |
|  | MeanD PORH | -0.168 | -0.143 | -0.171 | -0.238 | -0.207 | -0.087 | -0.052 |
|  | ΔMeanD | -0.151 | -0.01 | -0.127 | -0.205 | -0.216 | -0.135 | -0.095 |
|  |  | | | | | | | |
|  | MedD BL | -0.032 | -0.142 | -0.013 | -0.026 | -0.004 | 0.052 | 0.077 |
|  | MedD PORH | -0.155 | -0.133 | -0.161 | -0.227 | -0.205 | -0.089 | -0.059 |
|  | ΔMedD | -0.153 | -0.026 | -0.128 | -0.206 | -0.210 | -0.135 | -0.116 |
|  |  | | | | | | | |
|  | MeanL BL | -0.138 | -0.282 | -0.053 | 0.035 | 0.057 | 0.083 | 0.089 |
|  | MeanL PORH | 0.018 | -0.085 | 0.005 | 0.053 | 0.04 | 0.109 | 0.093 |
|  | ΔMeanL | 0.168 | 0.198 | 0.068 | 0.052 | 0.015 | 0.049 | 0.052 |
|  |  | | | | | | | |
|  | MedL BL | -0.159 | -0.303 | -0.116 | -0.098 | -0.026 | 0.114 | 0.062 |
|  | MedL PORH | -0.08 | -0.214 | -0.036 | -0.049 | -0.074 | 0.051 | -0.008 |
|  | ΔMedL | 0.006 | -0.036 | 0.039 | 0.014 | -0.074 | -0.06 | -0.03 |
|  |  | | | | | | | |
|  | BD BL | 0.055 | 0.206 | -0.006 | -0.061 | -0.077 | 0.036 | 0.083 |
|  | BD PORH | -0.033 | 0.118 | -0.069 | -0.188 | -0.203 | -0.157 | -0.126 |
|  | ΔBD | -0.079 | 0.012 | -0.035 | -0.116 | -0.15 | -0.178 | -0.200 |
|  |  | | | | | | | |
|  | MeanT BL | 0.09 | 0.273 | 0.1 | 0.067 | 0.068 | 0.027 | -0.019 |
|  | MeanT PORH | 0.302 | 0.261 | 0.144 | 0.350 | 0.369 | 0.230 | 0.061 |
|  | ΔMeanT | 0.228 | 0.052 | 0.062 | 0.266 | 0.301 | 0.213 | 0.075 |
|  |  | | | | | | | |
|  | FD BL | 0.117 | 0.335 | -0.008 | -0.046 | -0.056 | 0.014 | 0.016 |
|  | FD PORH | 0.078 | 0.263 | -0.021 | -0.059 | -0.064 | -0.016 | -0.048 |
|  | ΔFD | -0.057 | -0.064 | 0.018 | 0.015 | 0.007 | -0.069 | -0.12 |
|  |  | | | | | | | |
|  | FD error BL | -0.034 | 0.054 | 0.103 | 0.054 | -0.004 | -0.103 | -0.084 |
|  | FD error PORH | 0.179 | 0.009 | 0.181 | 0.219 | 0.168 | 0.131 | 0.148 |
|  | ΔFD error | 0.182 | -0.067 | 0.073 | 0.167 | 0.155 | 0.215 | 0.231 |
|  |  | | | | | | | |
|  | Target | 0.202 | 0.280 | 0.076 | 0.320 | 0.359 | 0.221 | 0.2 |

**Table S6.** **Kendall Tau correlation coefficients between demographic risk factors and microvascular metrics for foot in healthy people**. VAD – vessel area density; VLD – vessel length density; MeanD – mean diameter; MedD – median diameter; MeanL – mean length; MedL: - median length; BP – branchpoint density; MeanT – mean tortuosity; FD – fractal dimension; BL – baseline; PORH – post-occlusive reactive hyperaemia; Light green shading – correlation is significant at the 0.05 level (2-tailed); Dark green shading – correlation is significant at the 0.01 level (2-tailed).

| **Study participant characteristics** | **Healthy** | **T2DM** | **p-value** |
| --- | --- | --- | --- |
| Number | 11 | 11 |  |
| Sex [male/female] | 6/5 | 8/3 |  |
| Age [year] | 60 (9) | 60 (9) | 0.963 |
| Height [m] | 1.74 (0.08) | 1.75 (0.13) | 0.760 |
| Weight [kg] | 79 (15) | 93 (22) | 0.117 |
| BMI [kg/m^2^] | 26 (4) | 30 (5) | 0.062 |
| Systolic blood pressure [mmHg] | 120 | 129 | 0.192 |
| Diastolic blood pressure [mmHg] | 73 | 78 | 0.232 |
| HbA1c [mmol/mol] | N/A | 47 (4) |  |
| Metformin [n] | 0 | 5 |  |
| Sulphonylurea [n] | 0 | 2 |  |
| ACE/ARB [n] | 0 | 6 |  |
| Statin [n] | 0 | 4 |  |
| Ezetimibe [n] | 0 | 2 |  |
| BB [n] | 0 | 3 |  |
| Alphablocker [n] | 0 | 3 |  |

**Table S7.** **Study participant characteristics**: T2DM group and age- and parameter-matching control group. N/A: not applicable.

|  | **Hand** | | | **Foot** | | | **Hand vs Foot**  **Healthy** | **Hand vs Foot**  **T2DM** |
| --- | --- | --- | --- | --- | --- | --- | --- | --- |
| **Metric** | **Healthy** | **T2DM** | **p-value** | **Healthy** | **T2DM** | **p-value** | **p-value** | **p-value** |
| VAD [%] BL | 41 (11) | 32 (7) | **0.036** | 32 (7) | 38 (9) | 0.075 | **0.035** | 0.076 |
| VAD [%] PORH | 60 (9) | 50 (8) | **0.021** | 44 (9) | 46 (7) | 0.565 | **<0.001** | **0.016** |
| ΔVAD [%] | 55 | 62 | 0.684 | 42 | 27 | 0.311 | 0.440 | **0.030** |
|  | | | | | | | | |
| VLD [%] BL | 2 (0.6) | 2 (0.4) | 0.051 | 2 (0.5) | 2 (0.6) | 0.067 | 0.092 | **0.036** |
| VLD [%] PORH | 3 (0.3) | 3 (0.4) | 0.071 | 3 (0.5) | 3 (0.4) | 0.263 | **0.003** | 0.521 |
| ΔVLD [%] | 48 | 62 | 0.488 | 41 | 26 | 0.360 | 0.709 | **0.033** |
|  | | | | | | | | |
| MeanD [μm] BL | 52 (5) | 47 (4) | **0.026** | 44 (3) | 43 (3) | 0.373 | **<0.001** | **0.019** |
| MeanD [μm] PORH | 57 (7) | 53 (4) | 0.123 | 53 (4) | 45 (5) | **0.001** | 0.120 | **<0.001** |
| ΔMeanD [%] | 11 | 13 | 0.555 | 19 | 5 | **0.003** | 0.063 | 0.071 |
|  | | | | | | | | |
| MedD [μm] BL | 53 (6) | 48 (4) | **0.022** | 45 (3) | 44 (3) | 0.610 | **<0.001** | **0.038** |
| MedD [μm] PORH | 59 (8) | 55 (5) | 0.093 | 55 (5) | 46 (5) | **0.001** | **0.104** | **<0.001** |
| ΔMedD [%] | 12 | 15 | 0.557 | 22 | 5 | **0.002** | **0.048** | **0.038** |
|  |  |  |  |  |  |  |  |  |
| MeanL [μm] BL | 240 (20) | 240 (27) | 0.957 | 229 (16) | 223 (8) | 0.318 | 0.170 | 0.070 |
| MeanL [μm] PORH | 245 (16) | 246 (14) | 0.897 | 249 (16) | 237 (24) | 0.166 | 0.554 | 0.278 |
| ΔMeanL [%] | 3 | 3 | 0.873 | 9 | 6 | 0.427 | 0.079 | 0.509 |
|  | | | | | | | | |
| MedL [μm] BL | 179 (14) | 170 (9) | 0.076 | 167 (6) | 165 (4) | 0.337 | **0.020** | 0.156 |
| MedL [μm] PORH | 191 (15) | 182 (11) | 0.139 | 182 (13) | 175 (9) | 0.122 | 0.162 | 0.107 |
| ΔMedL [%] | 7 | 8 | 0.709 | 9 | 6 | 0.157 | 0.257 | 0.510 |
|  | | | | | | | | |
| BD [nodes/mm] BL | 2.7 (1) | 2 (0.3) | **0.034** | 2 (0.4) | 2.4 (0.6) | 0.094 | **0.034** | 0.081 |
| BD [nodes/mm] PORH | 4.4 (1) | 3.4 (0.7) | **0.012** | 3 (0.6) | 2.9 (0.6) | 0.764 | **0.001** | 0.125 |
| ΔBP [%] | 70 | 70 | 0.938 | 53 | 30 | 0.122 | 0.270 | **0.019** |
|  | | | | | | | | |
| MeanT [1] BL | 0.13 (0.01) | 0.13 (0.01) | 0.391 | 0.14 (0.01) | 0.14 (0.01) | 0.139 | 0.077 | **0.027** |
| MeanT [1] PORH | 0.14 (0.02) | 0.14 (0.01) | 0.395 | 0.14 (0.01) | 0.15 (0.01) | **0.037** | 0.409 | **0.018** |
| ΔMeanT [%] | 6 | 8 | 0.780 | 5 | 7 | 0.482 | 0.813 | 0.773 |
|  | | | | | | | | |
| FD [1] BL | 1.40 (0.03) | 1.39 (0.02) | 0.236 | 1.39 (0.03) | 1.42 (0.03) | **0.047** | 0.413 | **0.014** |
| FD [1] PORH | 1.45 (0.02) | 1.43 (0.02) | 0.846 | 1.41 (0.03) | 1.44 (0.01) | 0.052 | **0.004** | 0.572 |
| ΔFD [%] | 3 | 3 | 0.958 | 1.70 | 1.40 | 0.707 | 0.082 | **0.024** |

**Table S8.** **OCTA-derived metrics in well-controlled T2DM and matched-control groups**. Values are reported as mean (standard deviation). VAD – vessel area density; VLD – vessel length density; MeanD – mean diameter; MedD – median diameter; MeanL – mean length; MedL – median length; BD – branchpoint density; MeanT – mean tortuosity; FD – fractal dimension; [1] – dimensionless metric; fields highlighted in green show p<0.05 for paired t-test: baseline vs maximum: **bolded** p<0.05.

| **Imaging site** | **Metrics** | **Area under the ROC curve** | **Std. Error** | **Asymptotic significance level** | **Asymptotic 95% Confidence Interval** | |
| --- | --- | --- | --- | --- | --- | --- |
|  |  |  |  |  | Lower Bound | Upper Bound |
| **Hand** | FDerror PORH | 0.826 | 0.096 | 0.001 | 0.638 | 1.015 |
|  | FDerror BL | 0.81 | 0.105 | 0.003 | 0.605 | 1.015 |
|  | VAD BL | 0.752 | 0.115 | 0.028 | 0.528 | 0.977 |
|  | BD BL | 0.752 | 0.115 | 0.028 | 0.528 | 0.977 |
|  | VAD PORH | 0.719 | 0.118 | 0.064 | 0.487 | 0.951 |
|  | MedD BL | 0.711 | 0.116 | 0.069 | 0.483 | 0.938 |
|  | VLD BL | 0.702 | 0.117 | 0.083 | 0.473 | 0.932 |
|  | MeanD BL | 0.702 | 0.116 | 0.081 | 0.475 | 0.93 |
|  | BD PORH | 0.702 | 0.124 | 0.101 | 0.46 | 0.945 |
|  | MedD PORH | 0.645 | 0.122 | 0.234 | 0.406 | 0.883 |
|  | FD PORH | 0.645 | 0.127 | 0.253 | 0.396 | 0.893 |
|  | VLD PORH | 0.636 | 0.13 | 0.293 | 0.382 | 0.891 |
|  | MedL BL | 0.62 | 0.125 | 0.339 | 0.374 | 0.865 |
|  | MeanD PORH | 0.612 | 0.126 | 0.376 | 0.364 | 0.859 |
|  | MedL PORH | 0.537 | 0.128 | 0.772 | 0.286 | 0.789 |
|  | FD BL | 0.479 | 0.131 | 0.875 | 0.222 | 0.737 |
|  | MeanT BL | 0.421 | 0.128 | 0.539 | 0.171 | 0.672 |
|  | ΔVLD | 0.388 | 0.126 | 0.374 | 0.142 | 0.635 |
|  | MeanT PORJ | 0.372 | 0.132 | 0.332 | 0.113 | 0.631 |
|  | ΔFDerror | 0.24 | 0.105 | 0.013 | 0.034 | 0.445 |
|  | ΔMedD | 0.174 | 0.089 | 0 | -0.002 | 0.349 |
|  | ΔMeanD | 0.157 | 0.085 | 0 | -0.009 | 0.323 |
|  | ΔVAD | 0 | 0 | 0 | 0 | 0 |
|  | MeanL BL | 0 | 0 | 0 | 0 | 0 |
|  | MeanL PORH | 0 | 0 | 0 | 0 | 0 |
|  | ΔMeanL | 0 | 0 | 0 | 0 | 0 |
|  | ΔMedL | 0 | 0 | 0 | 0 | 0 |
|  | ΔBD | 0 | 0 | 0 | 0 | 0 |
|  | ΔFD | 0 | 0 | 0 | 0 | 0 |
|  | ΔMeanT | 0 | 0 | 0 | 0 | 0 |
| **Foot** | MedD PORH | 0.818 | 0.091 | <0.001 | 0.64 | 0.997 |
|  | MeanD PORH | 0.802 | 0.096 | 0.002 | 0.613 | 0.99 |
|  | ΔMeanD | 0.785 | 0.106 | 0.007 | 0.578 | 0.992 |
|  | ΔMedD | 0.777 | 0.109 | 0.011 | 0.564 | 0.99 |
|  | FD BL | 0.645 | 0.123 | 0.238 | 0.404 | 0.885 |
|  | FD PORH | 0.645 | 0.122 | 0.234 | 0.406 | 0.883 |
|  | VLD BL | 0.628 | 0.125 | 0.307 | 0.382 | 0.874 |
|  | MeanL PORH | 0.603 | 0.133 | 0.437 | 0.343 | 0.864 |
|  | VAD BL | 0.595 | 0.125 | 0.449 | 0.349 | 0.841 |
|  | MedL PORH | 0.587 | 0.129 | 0.501 | 0.334 | 0.839 |
|  | ΔFDerror | 0.587 | 0.129 | 0.503 | 0.333 | 0.84 |
|  | BD BL | 0.579 | 0.13 | 0.545 | 0.324 | 0.833 |
|  | ΔBD | 0.554 | 0.133 | 0.686 | 0.293 | 0.814 |
|  | MeanT BL | 0.545 | 0.131 | 0.728 | 0.289 | 0.802 |
|  | ΔMedL | 0.529 | 0.129 | 0.822 | 0.277 | 0.781 |
|  | ΔVAD | 0.496 | 0.134 | 0.975 | 0.233 | 0.759 |
|  | ΔVLD | 0.471 | 0.128 | 0.821 | 0.22 | 0.722 |
|  | MeanT PORH | 0.463 | 0.14 | 0.79 | 0.189 | 0.737 |
|  | ΔMeanL | 0.455 | 0.131 | 0.729 | 0.198 | 0.712 |
|  | MeanD BL | 0.455 | 0.129 | 0.724 | 0.202 | 0.707 |
|  | FDerror PORH | 0.455 | 0.13 | 0.727 | 0.199 | 0.71 |
|  | VLD PORH | 0.43 | 0.125 | 0.575 | 0.184 | 0.675 |
|  | MedL BL | 0.413 | 0.126 | 0.491 | 0.166 | 0.66 |
|  | MeanL BL | 0.38 | 0.125 | 0.339 | 0.135 | 0.626 |
|  | FDerror BL | 0.298 | 0.115 | 0.078 | 0.073 | 0.522 |
|  | ΔMeanT | 0.298 | 0.115 | 0.078 | 0.073 | 0.522 |
|  | VAD PORH | 0.231 | 0.104 | 0.01 | 0.028 | 0.435 |
|  | MedD BL | 0.231 | 0.102 | 0.009 | 0.031 | 0.432 |
|  | BD PORH | 0 | 0 | 0 | 0 | 0 |
|  | ΔFD | 0 | 0 | 0 | 0 | 0 |

**Table S9.** **Results from the univariate logistic regression leave-one-out analysis: area under the ROC curve for demographic risk factors and microvascular metrics for hand and foot classifying between people with type 2 diabetes and healthy people**. VAD – vessel area density; VLD – vessel length density; MeanD – mean diameter; MedD – median diameter; MeanL – mean length; MedL – median length; BP – branchpoint density; MeanT – mean tortuosity; FD – fractal dimension; BL – baseline; PORH – post-occlusive reactive hyperaemia. Green highlighted rows indicate area under the ROC curve greater than 0.7.
